# Serum growth differentiation factor-15 analysis as a malnutrition marker in hemodialysis patients

**DOI:** 10.3906/sag-2103-62

**Published:** 2021-08-30

**Authors:** Didem TURGUT, Deniz İlhan TOPCU, Cemile Cansu ALPEREN, Esra BASKIN

**Affiliations:** 1 Division of Nephrology, Department of Internal Medicine, Başkent University Ankara Hospital, Ankara Turkey; 2 Department of Biochemistry, Başkent University Ankara Hospital, Ankara Turkey; 3 Department of Internal Medicine, Başkent University Ankara Hospital, Ankara Turkey; 4 Division of Nephrology, Department of Pediatrics, Başkent University Ankara HospitalAnkara Turkey

**Keywords:** Malnutrition, nutrition, hemodialysis, growth differentiation factor-15, malnutrition/inflammation score (MIS)

## Abstract

**Background/aim:**

Growth differentiation factor (GDF)-15 is related to inflammation and mortality in many conditions. We aimed to determine if an elevated serum GDF-15 level is related to nutritional status of patients on hemodialysis (HD) and mortality.

**Materials and methods:**

Routine HD patients (n = 158) were included in the study and followed for 18 months. Some malnutrition/inflammation scoring indexes (malnutrition/inflammation score (MIS), controlling nutritional status (CONUT) score, and prognostic nutritional index (PNI)), biochemical parameters, and GDF-15 were used to build Cox regression multivariate models to study the association with mortality.

**Results:**

Among the patients, 90 (57 %) had a high MIS (≥8), which associates with worse status. The serum GDF-15 level was higher in the same group (p = 0.003). The serum GDF-15 level differentiated malnutrition/inflammation according to the MIS (p = 0.031). Age, GDF15, and C-reactive protein (CRP) were significantly associated with higher all-cause mortality risk. Patients with both age and GDF-15 above the mean had a hazard ratio of 2.76 (p = 0.006) when compared with those both < mean.

**Conclusion:**

In HD patients, the GDF-15 level is increased in worse nutritional status. Beyond the MIS, age, GDF-15 and CRP would be used together to estimate the worse clinical outcome in these patients.

## 1. Introduction

Growth differentiation factor-15 (GDF-15) is a TGF-β superfamily member and is widely expressed in mammalian tissues [1]. An elevated serum GDF-15 level is associated with numerous pathological conditions. Cardiovascular diseases [2], ineffective erythropoiesis [3], such chronic diseases as rheumatoid arthritis, end-stage renal failure, and diabetes [1], and many cancers [4] are associated with a high GDF-15 level. Notably, in cancer patients overexpressed GDF-15 was identified as a novel appetite regulator that causes anorexia and weight loss [5]. Weight loss, protein-energy wasting, and malnutrition/inflammation are the strongest predictors of over-all mortality in hemodialysis (HD) patients [68]. Some studies have examined the prognostic significance of GDF-15 in HD patients [9], but findings related to the correlation between GDF-15 and the severity of malnutrition/inflammation is inconsistent. Thus, the present study aimed to determine if there is an association between the GDF-15 level and nutrition parameters and malnutrition/inflammation scoring indexes in HD patients. An additional aim was to differentiate mortality according to HD patients’ malnutrition scores and serum GDF-15 levels.

-ly,

## 2. Materials and ethods

### Study design

This prospective observational study included patients with end-stage renal disease (ESRD) that were on conventional maintenance HD in our outpatient dialysis center. Patients were followed-up for 18 months. Inclusion criteria were >18 yearsage and undergoing HD for ≥4 h per session, ≥3 times per week, and for ≥12 months. To minimize confounding of inflammatory biomarkers, patients with active infection and those with a central venous catheter for vascular access during the previous 12 months were excluded. Patients with a history of any malignancy, inflammatory disease, or immunosuppressive drug usage were also excluded. To facilitate unbiased serum GDF-15 analysis patients with acute thrombosis or acute cardiovascular conditions (including acute coronary syndrome or acute heart failure) during the previous 12 months, known heart failure or an ejection fraction (EF) <55% were also excluded. Other exclusion criteria were unwillingness to participate in the study and a history of coronary artery bypass surgery. The study included 158 of 379 patients that met the inclusion criteria. 

### Data collection

All patients were assessed in terms of age, , cigarette smoking, dialysis dose (single-pool Kt/V) and vintage, comorbidities, body mass index (BMI), electrocardiography findings, biochemical parameters, malnutrition parameters, and malnutrition scores. Patients that had not smoked cigarettes for the previous 12 months were considered non-smokers. 

genderBlood samples were obtained immediately before the first midweek dialysis session of the month. Blood specimens (8 mL) were collected into gel tubes for GDF‑15, albumin, creatinine, blood urea nitrogen (BUN), CRP, potassium, and total iron-binding capacity (TIBC) analysis. Serum samples were centrifuged at 1200 g for 10 min at room temperature. For GDF‑15 measurement centrifuged samples were aliquoted and then stored at −80 °C until analysis. The serum GDF‑15 level was determined via an enzyme-linked immunosorbent assay (ELISA; Cloud Clone, Houston, TX,) using a spectrophotometer optical density of 450 nm (Epoch, BioTek Instruments Inc., Winooski, VT, USA), with intra- and inter-assay CVs of <10% and <12%, respectively. The GDF-15 level was expressed as ng mL^−1^. Creatinine, BUN, TIBC, and potassium levels were measured using an Abbott Alinity c analyzer according to the manufacturer’s instructions (Abbott Park, IL, USA).

EDTA whole- blood samples were used for complete blood count (CBC). CBC analyses were completed within 1 h after venipuncture and performed using Abbott CELL DYN Ruby hematology analyzer (Abbott Diagnostics, IL, USD). Hemoglobin, platelet, and white blood cells (WBCs), including leukocyte differentials, were measured as part of CBC. The platelet to lymphocyte ratio (PLR) and neutrophil-lymphocyte ratio (NLR) values were calculated using a related CBC parameter. Normalized protein catabolic rate (nPCR) [10] and a nutritional risk index (NRI) were calculated [11]. 

### Malnutrition scores

The malnutrition-inflammation score (MIS) is a scoring system and used to assess nutritional status quantitatively, especially in HD patients [12]. The MIS consist of 4 parts, 10 components, each representing different aspects of malnutrition-inflammation. Medical history of patient, physical examination findings, BMI, and laboratory parameters are the parts evaluated. And some of 10 components of the MIS have 4 levels of severity, ranging from 0 (normal) to 4 (severely abnormal). The sum of all components ranges from 0 (normal) to 30 (severely malnourished); higher scores indicate more severe malnutrition-inflammation [13]. Patient medical history includes dietary intake, weight changes, gastrointestinal symptoms, functional capacity, and comorbidities. Physical examination is performed to detect muscle wasting and loss of subcutaneous fat. In laboratory assessment, serum albumin and serum TIBC levels are measured. The MIS for each patient was recorded at initiation of the study. Clinical data, including age, , duration of dialysis, body weight and height, and the presence of comorbid conditions were obtained via the medical records. One of the investigators evaluated all the patients to assess physical morbidity, and subcutaneous muscle mass. After the assessment the patients were classified according to quartiles as follows: quartile 1 (MIS 0gender,-4 [n = 19]; quartile 2 (MIS 57 [n = 49]); quartile 3 (MIS ≥8 [n = 90]). Kalantar-Zadeh et al. [12] reported that the hazard ratio for death is almost 7-fold higher in those with an MIS ≥8 than the <8 ones (adjusted HR: 6.82; < 0.001) [12]. As such, the present study classified patients as low-risk of malnutrition (quartiles 1 and 2: score 0-7 [n = 68]) and high-risk of malnutrition (quartile 3: score ≥8 [n = 90]). 

-PThe prognostic nutritional index (PNI) was calculated as following: PNI = 10 × serum albumin concentration in g dL^−1^ + 0.005 × total lymphocyte count per mm^3^. Patients with a PNI >38 were considered normal, whereas those with a PNI of 3538 were considered to have a moderate risk of malnutrition, and those with a PNI <35 were considered to have a severe risk of malnutrition [14]. The controlling nutritional status (CONUT) score consists of serum albumin and total cholesterol levels, and the total lymphocyte count. Patients with CONUT scores of 01 have a normal nutritional status, whereas those with CONUT scores of 2--4 have a mild risk of malnutrition, those with CONUT scores of 58 have a moderate risk of malnutrition, and those with CONUT scores of 912 have a severe risk of malnutrition [15]. 

### Follow-up and end points

The HD patients were evaluated between March 2019 and October 2020, and mortality was verified until October 2020 (for 18 months). None of patients underwent renal transplantation or were transferred to another facility.

### Statistical analysis

Categorical variables are presented as number and percentage. Data with normal distribution are shown as mean ± SD and data not normally distributed are shown as median and interquartile range. For categorical variables the chi-square test was used. The independent samples t-test or MannWhitney U test was used for continuous variables. Variables with skewed distribution were logarithmically (log) transformed before further statistical analysis. Analysis of the correlation of each parameter was performed using Pearson’s or Spearman’s correlation coefficients. Results are given as correlation coefficient (r) and p values. 

-Differentiation of patients that have malnutrition/inflammation according to serum GDF-15 level was performed using the area under a receiver operating characteristic (AUROC) curve. The reference point was accepted for MIS≥8. AUROC was also made to determine the cut-off value of GDF-15 to predict mortality at the end of 18th month. An area under curve (AUC) of 0.5 indicates no predictive ability, whereas a value of 1 represents perfect predictive ability. Cut-off points were calculated by obtaining the best Youden index. 

The univariate Cox regression analysis was performed to investigate the independence of risk factors associated with all-cause mortality. If hazard ratios (HRs) for the variables in the univariate analysis were significant, we selected the covariates into the multivariable regression model. GDF-15 was analyzed as a continuous variable. A KaplanMeier curve censored for the high GDF-15 levels and MIS≥8 events with a log-rank test served for the cumulative recurrence rate of mortality at the end of 18th month. The cumulative survival probability was presented by graphical methods. Patients were further cross-classified according to GDF-15 (mean-=49.5) and age (mean=63.3) in three groups: those with both GDF-15 and age ≥ mean, those with both GDF-15 and age < mean (reference category), and those with only one of age or GDF-15 above the mean (age ≥ mean but not GDF-15 and GDF-15 ≥ mean but not age). Independent association with mortality was also studied for this dummy-coded variable.

The level of statistical significance was set at p < 0.05. Data were analyzed using IBM SPSS Statistics for Windows v.25.0 (IBM Corp., Armonk, NY).

## 3. Results

Among the 158 patients that met the eligibility criteria, 60 (37.9 %) were female. Median age of the study population was 66 years (1987 years). The median (IQR) and range of the serum GDF-15 values were 42.78 ng mL-^–1^ (24.7465.25 ng mL-^–1^), and 4.47183.90 ng mL-^–1^, respectively. Mean MIS was 8.51 ± 3.40 for the entire study group. The mean PNI was 38.11 ± 2.88 and 50% of the patients had moderate to severe malnutrition (PNI ≤38). The median CONUT score was 2 (07) and 51.9% of the patients had mild to moderate malnutrition (CONUT score: 2-8). None of the patients were MIS quartile 4 or had a PNI <35 or CONUT score of 9-12as such none of the patients had severe malnutrition. 

-,; ,The study population was divided into 2 groups regarding malnutrition/inflammation status according to the MIS. In total, 68 (43%) of the patients had a low MIS (quartiles 1 and 2; MIS score <8) and 90 (57%) had a high MIS (quartile 3; MIS ≥8). The PNI was lower and the CONUT score was higher in the patients with a high MIS (p = 0.001 and p = 0.024, respectively). The serum GDF-15 level was significantly higher in the patients with a high MIS than in those with a low MIS (37.42 vs. 47.82, p = 0.003). Logarithmic transformation of the GDF-15 data (logGDF-15) showed that the difference was still significant according to parametric statistical tests. As nutritional parameters, NRI and nPCR were also lower in the patients with a high MIS (p = 0.023 and p = 0.001, respectively). In addition, the serum albumin, serum creatinine, and BUN levels were lower in the patients with a high MIS. The inflammatory parameters CRP and NLR were slightly higher in the patients with a high MIS, as well, but only PLR was significantly higher (p = 0.021). In all, 18.9% (n = 30) of the patients died during the 18-month follow-up period. Demographic data, laboratory results, and nutritional parameters are summarized according to low and high MIS in Table 1. 

**Table 1:** Demographic findings, laboratory results and nutritional status findings of study group.

	MIS Score < 8(Quartile 1 and 2; n = 68)	MIS Score ≥ 8(Quartile 3; n = 90)	p
Demographic			
Age (years)	63 (IQR:17) (Mean R:67.71)	69.50 (IQR:16) (Mean R:88.41)	0.005
Women (n)	23 (14.6%)	37 (23.4%)	0.409
Body mass index (kg/m2)	26.71 ± 5.59	25.19 ± 5.40	0.086
Cigarette smoking (n)	13 (8.2 %)	17 (10.8 %)	0.564
Diabetes mellitus (n)	22 (13.9 %)	40 (25.3 %)	0.084
Coronary artery disease (n)	34 (21.5 %)	57 (36.1 %)	0.065
Hypertension (n)	58 (36.7 %)	77 (48.7 %)	0.569
Left ventricular hypertrophy (n)	60 (38.1 %)	83 (52.2 %)	0.282
Diastolic dysfunction (n)	48 (30.4 %)	76 (48.1 %)	0.029
Dialysis vintage (months)	36 (IQR:43) (Mean R:67.26)	63 (IQR:89) (Mean R:88.74)	0.003
Dialysis dose (Kt/V single pool)	1.54 ± 0.273	1.57 ± 0.262	0.425
Biochemical measurements			
Hemoglobin (g/dL)	11.54 ± 1.32	11.23 ± 1.22	0.132
Serum albumin (g/dL)	3.91 ± 0.23	3.72 ± 0.29	0.001
Serum creatinine (mg/dL)	8.86 ± 0.30	8.02 ± 0.23	0.026
BUN (mg/dL)	76.76 ± 17.80	67.13 ± 18.10	0.001
Serum potassium	5.10 ± 0.64	4,95 ± 0.69	0.172
Total iron binding capacity (mg/dL)	148.94 ± 47.90	131.34 ± 46.30	0.021
Total cholesterol (mg/dL)	173.12 ± 35.93	164.38 ± 37.37	0.141
C-reactive protein (mg/l)	18.56 ± 4.12	20.78 ± 3.27	0.674
NLR	3.177 ± 1,93	3.59 ± 3.03	0.297
PLR	126.10 ± 55.28	153.17 ± 89.50	0.021
Nutrition parameters			
nPCR (nPNA) (g.kg–1.day–1)	1.067 ± 0.239	0.978±0.235	0.001
NRI	100.69 ± 3.83	97.48 ± 4.32	0.023
PNI score	39.22 ± 2.33	37.26 ± 2.67	0.001
PNI score ≤ 38 (n)	20 (12.7 %)	59 (37.3 %)	0.001
CONUT score (Mild and moderate malnutrition; 2–8) (n)	28 (17.7%)	54 ± (34.2%)	0.024
GDF-15 (ng/mL)	37.42 (IQR:33.54) (Mean R:70.46)	47.82 (IQR:47.95) (Mean R:86.33)	0.003
LogGDF-15	1.58 ± 0.28	1.64 ± 0.30	0.023
Death (n)	8 (11.8 %)	22 (24.4 %)	0.034
Time to death (months)	16.50 (IQR:8) (Mean R:17.50)	14.00 (IQR:4) (Mean R:14.77)	0.475

Results are presented as mean ± standard deviation (95% confidence interval) and median (IQR: interquartile range) for continuous variables and n (%) for categorical variables. BUN: Blood urea nitrogen; NLR: neutrophil-to-lymphocyte ratio; PLR: platelet-to-lymphocyte ratio; nPCR: normalized protein catabolic rate; NRI: nutrition risk index; PNI: prognostic nutritional index; CONUT: The Controlling Nutritional Status; MIS: malnutrition-inflammation score; GDF-15: growth differentiation factor-15; logGDF-15: logarithmically transformed Growth Differentiation Fcator-15; Mean R: mean rank.

Based on correlation analysis of logGDF-15 with other serum biomarkers of malnutrition/inflammation, there was a negative correlation between the serum creatinine, serum potassium, and serum albumin levels. Additionally, logGDF-15 was positively correlated with CRP and logarithmic transformation of age (logAge). There weren’t any significant correlations between logGDF-15, and other nutrition parameters or biochemical measurements. The correlations data are summarized in Table 2. 

**Table 2:** Correlation of laboratory tests and nutritional scores with logarithmically transformed Growth Differentiation Factor-15.

	r	p		r	p
logAge	0.222	0.005	nPCR	0.118	0.144
BMI	0.094	0.242	NRI	0.154	0.054
logDvintage	0.045	0.577	NLR	0.055	0.494
Serum albumin	–0.415	0.018	PLR	0.134	0.194
Serum creatinine	–0.215	0.007	MIS	0.407	0.009
Serum potassium	–0.207	0.009	PIN score	–0.191	0.016
C-reactive protein	0.183	0.021	CONUT Score	0.268	0.001

BMI: Body Mass Index; CONUT: The Controlling Nutritional Status; MIS: malnutrition-inflammation score; logGDF-15: logarithmic transformation of growth differentiation factor-15; logDvintage: logarithmic transformation of dialysis vintage; NLR: neutrophil-to-lymphocyte ratio; PLR: platelet-to-lymphocyte ratio; PNI: prognostic nutritional index; logAge: logarithmic transformation of age.

**Figure 1:**
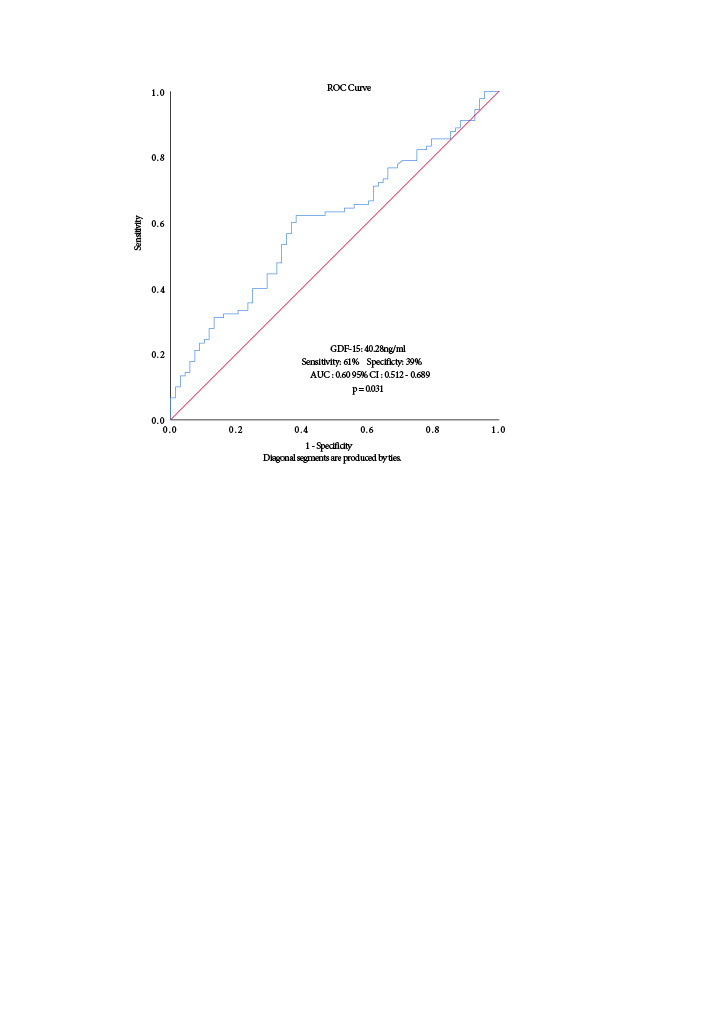
ROC curve from serum GDF-15 to predict malnutrition/inflammation according to MIS

**Figure 2:**
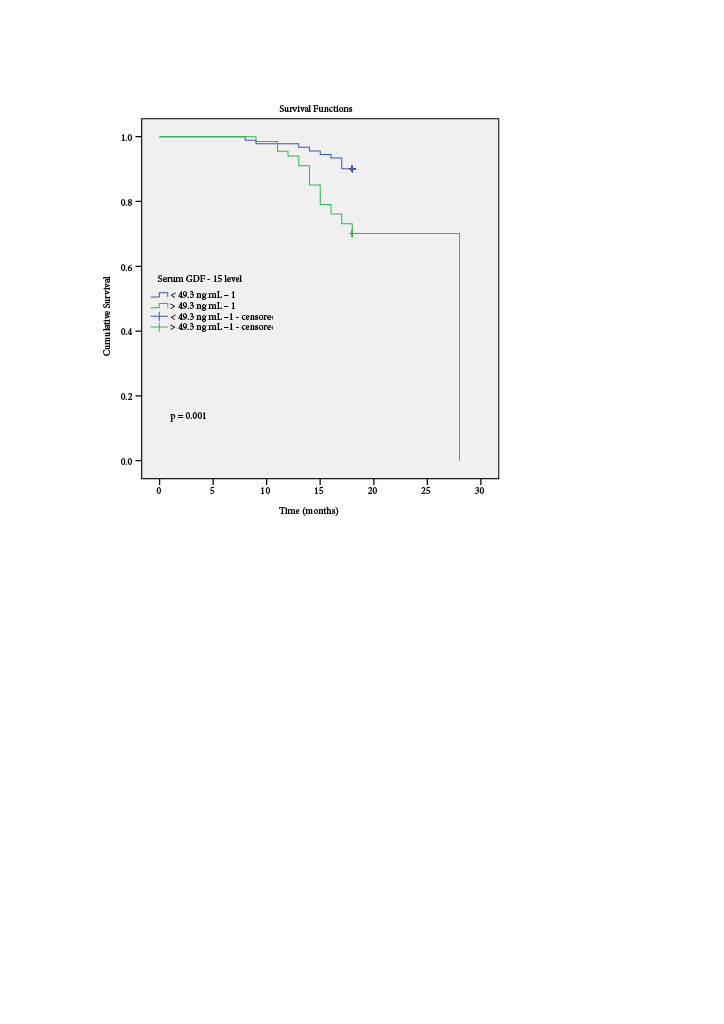
The Kaplan-Meier survival curves to predict mortality after 18 months according to serum GDF-15 level

**Figure 3:**
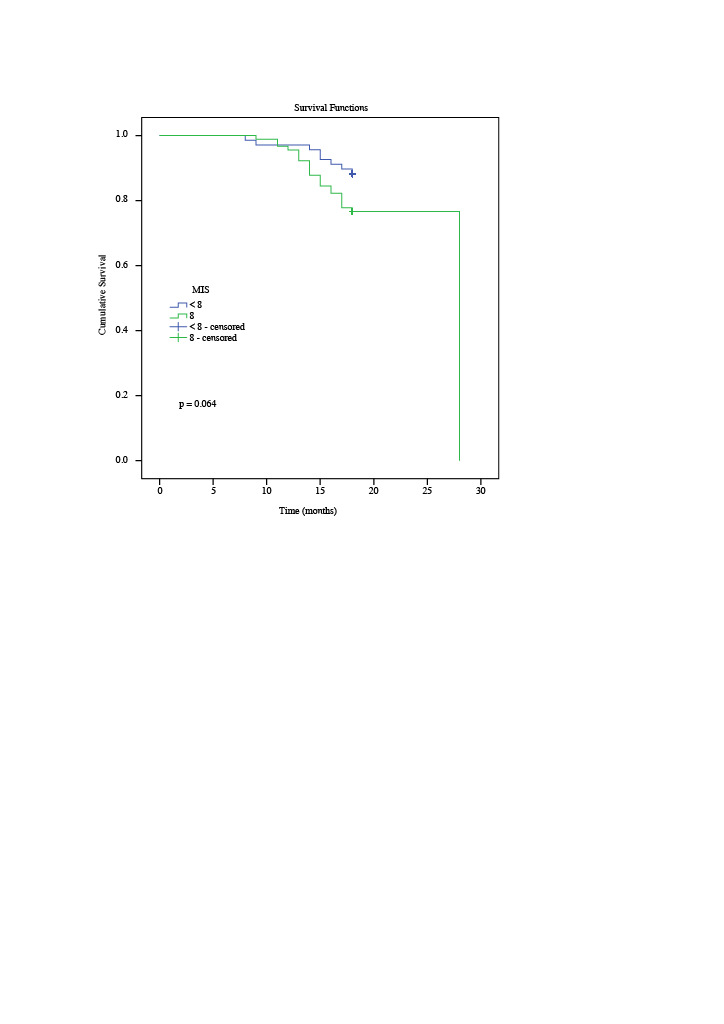
The Kaplan-Meier survival curves to predict mortality after 18 months according to MIS

Area under a receiver operating characteristic (AUROC) was performed to explore the ability of the serum GDF-15 level to predict malnutrition according to the MIS, PNI, and CONUT scores. The serum GDF-15 level significantly differentiated malnutrition/inflammation according to MIS (AUROC: 0.60; 95% CI: 0.5120.689; p-= 0.031). The cutoff value for GDF-15 was 40.28 ng mL^–1^ with sensitivity of 61% and specificity of 39% for predicting malnutrition (Figure 1). AUROC curves for the ability of GDF-15 to predict malnutrition according to the PNI and CONUT score were not significant (AUROC: 0.428; 95% CI: 0.3390.517; p = 0.117 and AUC: 0.433; 95% CI: 0.3440.523; 0.149, respectively).

ther AUROC curves were also performed to explore the reference points for the Meier survival analysis (Log Rank method) of the MIS and the serum GDF-15 levels to predict mortality after 18 months. The GDF-15 cutoff value was 49.3 ng mL--p=AnoKaplan ^–^1, with sensitivity of 70% and specificity of 39% for predicting mortality (AUROC: 0.709; 95% CI: 0.5910.829; p=0.003). The MIS cut-off value was 8, with sensitivity of 73% and specificity of 53% for predicting mortality (AUROC: 0.620; 95% CI: 0.502-0.737; 0.042). Figure 2 and Figure 3 show the Kaplan–Meier survival curves in patients with GDF-15 > 49.3 ng mL-p=^–^1 and those with MIS≥8 values. Meier analysis showed a significantly lower event-free survival time in the high GDF-15 group than the low GDF-15 group (17.6 months vs. 23.9 months, Kaplan og ank = 0.001, Figure 2). But the Meier analysis of event-free survival time difference between high and low MIS was not statistically different (17.5 months vs. 24.8 months, og ank = 0.064, Figure 3).

Univariate and multivariate analysis results for all-cause mortality are shown in Table 3. The variables entering the univariate analysis were age,LRPKaplan LRP dialysis vintage, serum albumin, C-reactive protein, serum creatinine, NLR, PLR, nPCR (nPNA), serum GDF-15, MIS (MIS≥8), PNI score (PNI >38), and CONUT score (28). In the multivariate Cox regression, a final statistically significant Model-1 was built with GDF-15, age, and C-reactive protein. Baseline serum GDF-15 (HR 1.052, 95% CI 1.0061.115, = 0.001), age (HR 1.068, 95% CI 1.026-1.112, = 0.001), and C-reactive protein (HR 1.008, 95% CI 1.0021.012, P = 0.049) were all independent predictors for 18th mortality. To combine both age and GDF-15 affect in predicting mortality--PP- we created multivariate Cox regression Model-2. Patients with both age and GDF-15 above the mean had a HR of 18-month death of 2.76 (95% CI 1.375.02, 0.006) when compared with the reference category (both age and GDF-15 below mean). Patients with only one of the variables above the mean had a hazard ratio of 18-month mortality of 1.16 (1.0021.816, 0.02) when compared with the reference category. -p=-p=

**Table 3:** Association of serum GDF-15 level and other variables with 18-month mortality: Univariate and multivariate model of cox regression analysis.

	Univariate Analysis		Multivariate Analysis	
Tested variables	HR (95% CI)	p	HR (95% CI)	p
Age (years)	1.073 (1.033–1.113)	0.003	1.068 (1.026-1.112)	0.001
Dialysis vintage (months)	0.997 (0.992–1.003)	0.352	-	-
Serum albumin (g/dL)	0.242 (0.073–0.800)	0.020	-	-
C-reactive protein (mg/l)	1.009 (1.003–1.014)	0.002	1.008 (1.002-1.012)	0.049
Serum creatinine (mg/dL)	0.778 (0.668–0.907)	0.001	-	-
NLR	1.132 (1.040–1.232)	0.004	-	-
PLR	1.004 (1.001–1.008)	0.024	-	-
nPCR (nPNA) (g.kg–1.day–1)	0.268 (0.055–1.313)	0.104	-	-
GDF-15 (ng/mL)	1.019 (1.011–1.027)	<0.001	1.052 (1.006-1.115)	0.001
MIS (MIS ≥ 8)	2.119 (0.938–4.785)	0.071	-	-
PNI score (PNI >38)	0.419 (0.191–0.920)	0.063	-	-
CONUT score (2–8)	0.889 (0.427–1.848)	0.752	-	-

GDF-15: growth differentiation factor-15; NLR: neutrophil-to-lymphocyte ratio; PLR: platelet-to-lymphocyte ratio; nPCR: normalized protein catabolic rate; PNI: prognostic nutritional index; CONUT: The Controlling Nutritional Status; MIS: malnutrition-inflammation score.

## 4. Discussion

The present study investigated the effectiveness of the serum GDF-15 for predicting malnutrition and all-cause mortality, as well as its correlation with nutrition/inflammation laboratory measurements in HD patients. The findings indicate that the serum GDF-15 level is useful for estimating the nutritional status of HD patients when other scoring systems are inconclusive. Assessment of the nutritional status is a routine part of the care of maintenance HD patients. Monthly dietary assessment, physical examination, and laboratory examination is the primary method used to estimate nutritional changes. MIS, originally developed by KalantarZadeh et al. [12], is a comprehensive tool for assessing the nutritional and inflammatory status and is associated with morbidity/mortality in HD patients. Newly used scores like PNI and CONUT are also informative about nutritional status of the patients [14,16]. none of the current studies analyzed these scores and biochemical parameters together. According to our findings, serum GDF-15 level with MIS is stronger than PNI and CONUT to estimate malnutrition in our HD patients. 

- But In the present study patients with a high MIS (≥8) were older and had a longer dialysis vintage than those with a low MIS as expected. Moreover, the serum albumin, serum creatinine, and BUN levels were lower in the patients with a lower nutritional status. The catabolic measurements nPCR and NRI were also lower in the patients with a high MIS, which is in agreement with the literature [11]. Inflammatory status assessment showed the CRP and NLR were similar in the patients with a high and low MIS, but that PLR was significantly higher in those with a high MIS, which is in agreement with Turkmen et al. [17]. They reported that PLR was superior to NLR for assessing inflammation in ESRD patients. All biochemical parameters directing worse nutritional and inflammatory status was correlated with our high risk patient group (MIS ≥8), highlighting the data related to malnutrition/inflammation process in HD patients, as reported earlier [18].

Other measures of malnutrition/inflammation, including the PNI and CONUT scores were compatible with the MIS. Some studies analyzed the PNI and CONUT score in HD patients, reporting that a lower PNI and higher CONUT scores were correlated with poorer outcomes [14, 19,20]. In mortality studies related to malnutrition/inflammation in HD patients, the MIS was observed to be a predictor of mortality and dialysis outcome [1221]. The PNI was independently associated with all-cause and CVD mortality in peritoneal dialysis patients [19]. The CONUT score was noted to be a clinical predictor of all-cause mortality in peritoneal dialysis patients [20]. In our study, the relationship between mortality , and the MIS, PNI, and CONUT score were analyzed together. First, AUROC curve analysis of GDF-15 level and MIS were significantly correlated with mortality at the end of the 18th month of follow-up. Nonetheless, the PNI and CONUT score did not significantly differentiate mortality, perhaps because the clinical assessment part of MIS makes the score stronger than the PNI and CONUT score in HD patients. Second, in the Meier survival analysis, it was documented that patients with high GDF-15 serum levels had worse event-free survival for 18 months. None of the scores (MIS, PNI, CONUT) were associated with significant ,Kaplan outcomes. Third, in the univariate Cox regression analysis of potential predictors, we did not find any significant mortality-related estimation associated with MIS, PNI, or CONUT scores. Since this time MIS was also excluded as an estimator, GDF-15 became more valuable in the patients with worse nutritional status. In the multivariate analysis final model built with GDF-15, age, and CRP which were significant predictors of mortality. Three studies analyzed GDF-15 and mortality in HD patients, and all reported that a high GDF-15 level was associated with a high risk of mortality [9,22,23]; however, none analyzed the patients’ malnutrition/inflammation status. Breit et al. [22] reported that in a Swedish study population a high GDF-15 level was associated with poor nutrition status, based on subjective global assessment questionnaires only. Our study expands upon the existing literature on GDF-15 in HD patients with malnutrition. The MIS together with the GDF-15 level might be considered a combined marker of the malnutrition/inflammation status and a good predictor for mortality in HD patients especially in the early period of malnutrition/inflammation. 

GDF-15 is a stress-induced cytokine and almost all tissues can express GDF-15 in response to various forms of stress [24]. It was recently reported that GDF15 activates a receptor composed of a GFRAL and RET dimer. This receptor group is specific to area postrema of the brain stem that is responsible from anorexia, vomiting, and nausea when it is activated [25]. Experimental data show that lack of GFRAL is associated with elevated GDF-15 and a decrease in food intake in mice [26]. Clinical data show that in healthy controls GDF-15 is positively correlated with the adiponectin level and negatively correlated with BMI and body fat mass [27]. In HD patients, exposure to multiple inflammatory agents, cardiac problems, and uremia might all be related to increased cellular stress, resulting in activation of GDF-15 and the nutrition axis. In the present study the GDF-15 level was significantly higher in the patients with a high MIS. In addition, the GDF-15 level was correlated with markers of nutrition and inflammation, such as low albumin, low serum potassium, and high CRP levels. GDF-15 was also more strongly correlated with the MIS other than PNI and CONUT scoreut GDF-15 was not correlated with BMI or HD vintage. 

In literature, outcomes of GDF-15 and mortality studies in HD patients are similar. In the latest study, Chang et al. demonstrated that HD patients with older age, higher plasma GDF-15 concentrations, and lower albumin levels are more likely to have greater risks for all-cause death [28]. In our final multivariate model,. B age, serum GDF-15, and CRP were independent risk factors in line with the literature. We also created a multivariate model combining age and GDF-15 to find out the unified effect of age and GDF-15 together. We found that both with increased age and high GDF-15 all-cause mortality. We think that this finding is valuable particularly in elderly HD patients is already difficult to obtain malnutrition/inflammation.

; increases by 2.76 times significantlywhich Nakajima et al. analyzed GDF-15 in patients before cardiovascular surgery and reported that the GDF-15 level helps identify the risk of muscle wasting and renal dysfunction before cardiovascular surgery [29]. Several mechanisms have been suggested to be related to GDF-15 induced muscle wasting [30]. As such, it is thought that an elevated GDF-15 level results in loss of appetite, anorexia, and cachexia, and directly affects muscle wasting and consequent weight loss [30]. GDF-15 might be useful for the objective assessment of malnutrition risk during the early period of HD, irrespective of weight loss or prominent inflammation. The present findings show that according to MIS, the GDF-15 level significantly differentiated malnutrition/inflammation with a cut-off 40.28 ng mL^–1^—this cut-off has not been previously reported. We excluded patients with all obvious infectious states, known cardiac problems, and inflammatory conditions to make GDF-15 reliable in the assessment of malnutrition, but all above finding needs to be confirmed by larger-scale studies. 

There are some noteworthy limitations of this study. Firstly, our study sample size was relatively small. Secondly, cross-sectional laboratory values and GDF15 levels might not reflect substantial intra-individual variability over time. Finally, GDF-15 cut-off points might change because of laboratory analysis techniques. 

In conclusion, the assessment of nutritional status in HD patients is complicated. The present findings indicate still that a high GDF-15 level is correlated with the MIS, even in moderately malnourished HD patients. Early detection of malnutrition/inflammation—before any marked cardiac or metabolic complications occur—especially in the older population could become the cornerstone for control of nutritional problems in HD patients, but additional research is required to confirm the present study’s findings. 
